# Commission in Lunacy

**Published:** 1848-01-01

**Authors:** 


### COMMISSION IN LUNACY.

### HENRY KEITH STEWART, ESQ.

*Commission executed Nov.* 19, 1847, *before* EDW. WINSLOW, Esq., *Master in Lunacy.*

Thirteen Jurors, (Common.)

MR. J. BAILY, Counsel for the Commission; Messrs. STONE & TURNER, Solicitors.

Edgar Barker, of Edgeware-road, surgeon, examined by Mr. BAILY.—Am a member of the College of Surgeons; know Mr. Henry Keith Stewart; have attended him professionally ten or twelve years. He had epileptic fits. The effect produced was gradual destruction of his mental capacity; it now amounts to total unsoundness of mind, and he is totally incapable of taking care of himself or his property.

By the COMMISSIONER.—I saw him on the occasion of his first seizure with epilepsy; have seen him every year during the last ten years, sometimes weekly. The fits were formerly about once a week, now they are sometimes several in a day. They produced a very gradual effect on his mind; his mind was very little influenced by the disease at first.

By Mr. BAILY.—On the 17th February last (1847), he was certainly in his present state of mind. I have seen him several times in the interval from that time, and he has been in the same state.

By the Commissioner.—I have not the least doubt that on the 17th February, 1847, he was of unsound mind, and incapable of taking care of himself or his property.

James York, surgeon, examined by Mr. Baily.—I reside at Windsor-terrace, Edgeware-road; am a surgeon; know Mr. Henry Keith Stewart; have known him for some years; first called in to attend him in May 1845; not been in the habit of attending him since; but in August last I attended him professionally; then he was in a state of unsound mind—quite idiocy; have not seen him since; have seen him about the garden, but had not seen him professionally this year except in August last; can speak confidently as to his state in August.

Rebecca Sorrell examined by Mr. Baily.—I know Mr. Henry Keith Stewart. He has been afflicted with epilepsy since he was twelve or thirteen years old. Twelve days' interval between the fits is the longest I have known, and that very rarely. He was incapable before 17th February last; he got worse every year. I have seen him to-day; his state of mind to-day is as usual.

By the Commissioner.—He very seldom speaks; now and then he speaks of his own accord, very incoherently, of his sister. The day before yesterday he said, "Bless my little sister, how I love her!" and then he forgot all about her. He never dresses himself, or feeds himself; I don't think he would ask for his food if he did not have it. He has had between 1500 and 1600 fits since he was twelve years old; he is now twenty-six years old. He has been as bad as he is now about two years; he has been very helpless several years—as bad as he is now two or three years. For three years he has not dressed himself.

Mr. Barker recalled by the Commissioner.—I have just seen Mr. Henry Keith Stewart; he is now exactly as I before stated; his mind is just the same.

Mr. York recalled by the Commissioner.—I have just seen Mr. Henry Keith Stewart; he is now quite in the same state as I before described.

The Commissioner and Jury visited Mr. Henry Keith Stewart in an adjoining room. He was sitting in an easy chair with a Testament before him. At first sight he appeared to be reading, but in fact he was not. He had a somewhat intelligent countenance, but he did not reply to any question.

*Verdict.*—A person of unsound mind, and not sufficient for the government of himself and his property, and that he had been in the same state from the 17th February, 1847.

